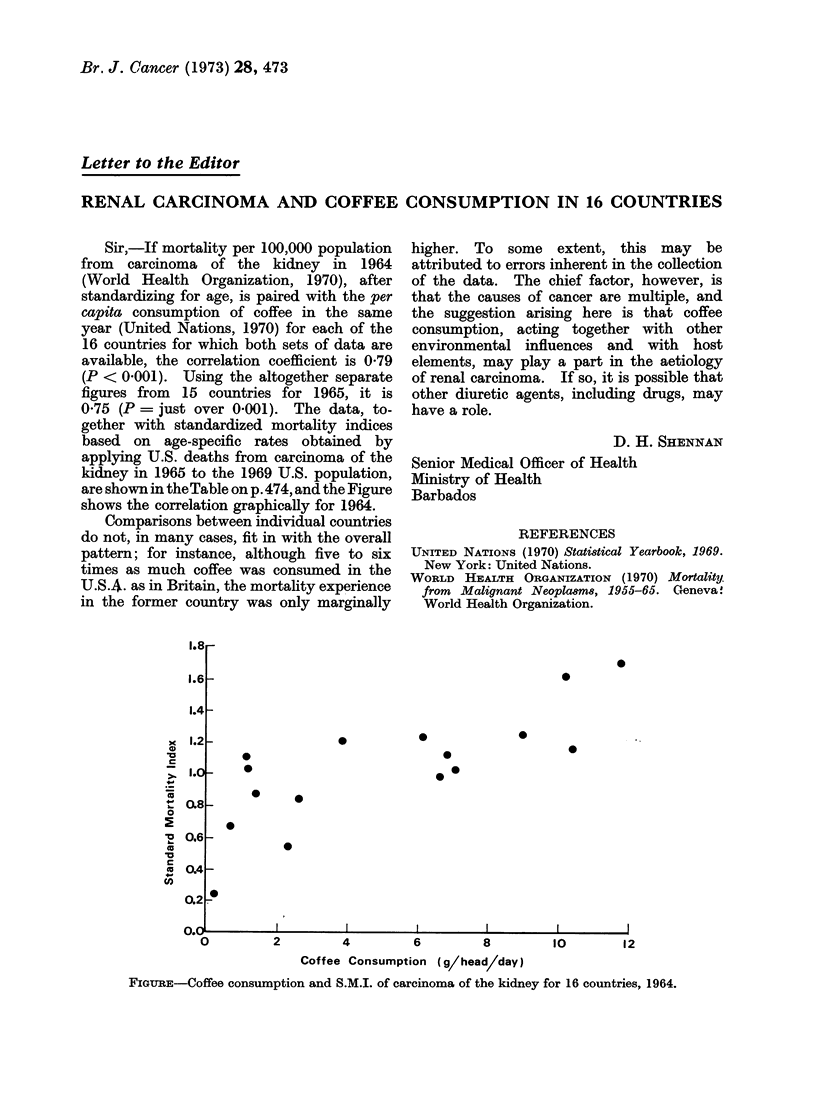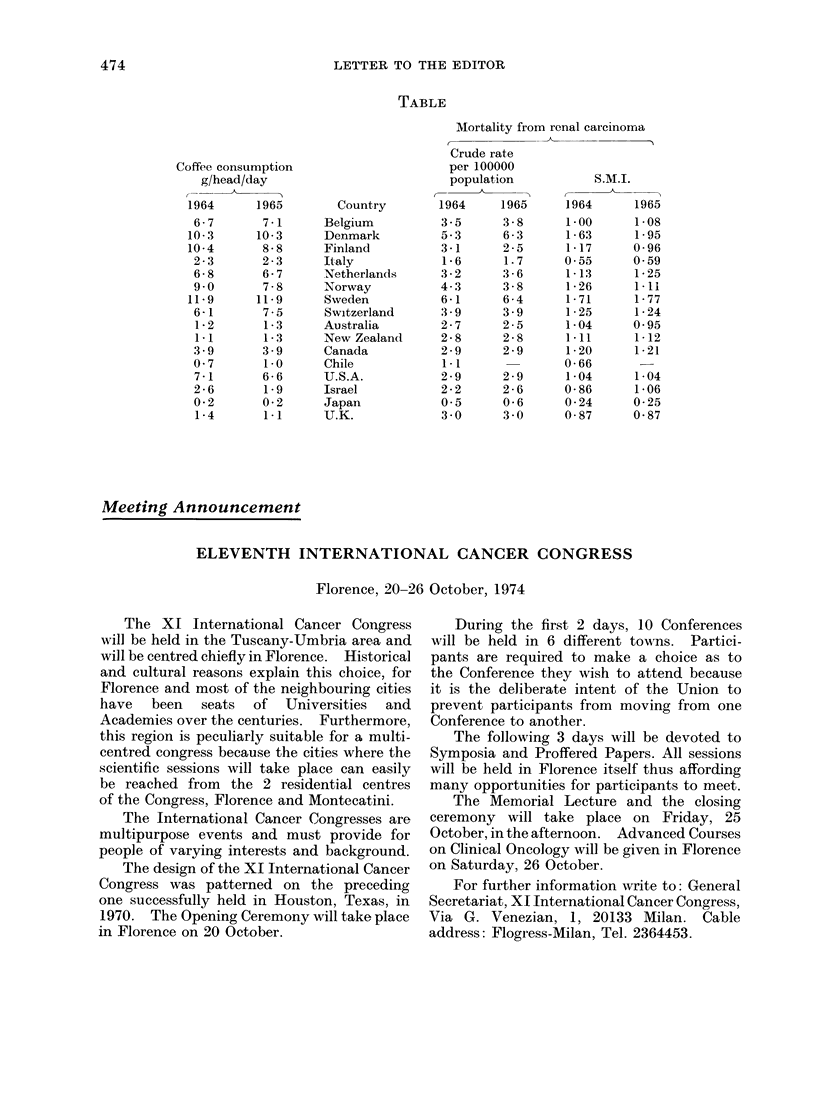# Letter: Renal carcinoma and coffee consumption in 16 countries.

**DOI:** 10.1038/bjc.1973.176

**Published:** 1973-11

**Authors:** D. H. Shennan


*Br. J. Cancer* (1973) **28**, 473

### *Letter to the Editor*

### RENAL CARCINOMA AND COFFEE CONSUMPTION IN 16 COUNTRIES

Sir,—If mortality per 100,000 population from carcinoma of the kidney in 1964 (World Health Organization, 1970), after standardizing for age, is paired with the *per capita* consumption of coffee in the same year (United Nations, 1970) for each of the 16 countries for which both sets of data are available, the correlation coefficient is 0·79 ($P < 0{\cdot}001$). Using the altogether separate figures from 15 countries for 1965, it is 0·75 ($P =$ just over 0·001). The data, together with standardized mortality indices based on age-specific rates obtained by applying U.S. deaths from carcinoma of the kidney in 1965 to the 1969 U.S. population, are shown in the Table on p. 474, and the Figure shows the correlation graphically for 1964.

Comparisons between individual countries do not, in many cases, fit in with the overall pattern; for instance, although five to six times as much coffee was consumed in the U.S.A. as in Britain, the mortality experience in the former country was only marginally higher. To some extent, this may be attributed to errors inherent in the collection of the data. The chief factor, however, is that the causes of cancer are multiple, and the suggestion arising here is that coffee consumption, acting together with other environmental influences and with host elements, may play a part in the aetiology of renal carcinoma. If so, it is possible that other diuretic agents, including drugs, may have a role.

D. H. Shennan

Senior Medical Officer of Health
Ministry of Health
Barbados

REFERENCES

United Nations (1970) *Statistical Yearbook, 1969.* New York: United Nations.

World Health Organization (1970) *Mortality from Malignant Neoplasms, 1955–65.* Geneva: World Health Organization.

Figure—Coffee consumption and S.M.I. of carcinoma of the kidney for 16 countries, 1964.

TABLE

| Coffee consumption g/head/day | | | Mortality from renal carcinoma: Crude rate per 100000 population | | S.M.I. | |
|---|---|---|---|---|---|---|
| 1964 | 1965 | Country | 1964 | 1965 | 1964 | 1965 |
| 6·7 | 7·1 | Belgium | 3·5 | 3·8 | 1·00 | 1·08 |
| 10·3 | 10·3 | Denmark | 5·3 | 6·3 | 1·63 | 1·95 |
| 10·4 | 8·8 | Finland | 3·1 | 2·5 | 1·17 | 0·96 |
| 2·3 | 2·3 | Italy | 1·6 | 1.7 | 0·55 | 0·59 |
| 6·8 | 6·7 | Netherlands | 3·2 | 3·6 | 1·13 | 1·25 |
| 9·0 | 7·8 | Norway | 4·3 | 3·8 | 1·26 | 1·11 |
| 11·9 | 11·9 | Sweden | 6·1 | 6·4 | 1·71 | 1·77 |
| 6·1 | 7·5 | Switzerland | 3·9 | 3·9 | 1·25 | 1·24 |
| 1·2 | 1·3 | Australia | 2·7 | 2·5 | 1·04 | 0·95 |
| 1·1 | 1·3 | New Zealand | 2·8 | 2·8 | 1·11 | 1·12 |
| 3·9 | 3·9 | Canada | 2·9 | 2·9 | 1·20 | 1·21 |
| 0·7 | 1·0 | Chile | 1·1 | — | 0·66 | — |
| 7·1 | 6·6 | U.S.A. | 2·9 | 2·9 | 1·04 | 1·04 |
| 2·6 | 1·9 | Israel | 2·2 | 2·6 | 0·86 | 1·06 |
| 0·2 | 0·2 | Japan | 0·5 | 0·6 | 0·24 | 0·25 |
| 1·4 | 1·1 | U.K. | 3·0 | 3·0 | 0·87 | 0·87 |